# Association of Number of Teeth with ADL/IADL in Korean Middle-Aged and Older Adults: An Analysis of the 7th Korean Longitudinal Study of Aging

**DOI:** 10.3390/ijerph191912840

**Published:** 2022-10-07

**Authors:** Nu-Ri Jun, Jae-Hyun Kim, Jong-Tae Park, Jong-Hwa Jang

**Affiliations:** 1 Department of Public Health, Graduate School, Dankook University, Cheonan-si 31116, Korea; 2 Department of Health Administration, College of Health Science, Dankook University, Cheonan-si 31116, Korea; 3 Department of Oral Anatomy, College of Dentistry, Dankook University, Cheonan-si 31116, Korea; 4 Department of Dental Hygiene, College of Health Science, Dankook University, Cheonan-si 31116, Korea

**Keywords:** age, ADL, IADL, dentistry, natural teeth, dental implant, middle-aged adult, older adult

## Abstract

We determined the association between the number of natural and implant teeth with activities of daily living (ADL) and instrumental ADL (IADL) levels in middle-aged and older adults aged ≥ 55 years. We included 6,925 participants, who underwent a computer-assisted personal interview in the 7th Korean Longitudinal Study of Aging. After controlling for general characteristics, the associations between the number of natural and implant teeth with ADL and IADL levels were examined using multiple regression analysis. The participants had 21.2 natural teeth and 1.08 implant teeth on average. The ADL and IADL levels were 0.61 and 1.56, 0.40 and 1.16, and 1.10 and 0.31 in participants with ≤ 9, 10–19, and ≥ 20 teeth, respectively. There was no significant association between ADL and the number of natural and implant teeth (*p* > 0.05). However, a one-unit increase in IADL score was associated with a reduced number of natural (β = −0.031, *p* < 0.001) and implant (β = −0.194, *p* = 0.006) teeth. Difficulties regarding IADL were associated with fewer natural and implant teeth, suggesting that maintaining and managing the number of teeth is critical to promoting the health of middle-aged and older adults.

## 1. Introduction

The population of older adults aged ≥ 65 years accounted for 15.7% of the total population in 2020 in South Korea and is predicted to reach 20.3% by 2025, with the country becoming a superaged society. Estimates suggest that, by 2047, approximately half (49.6%) of all households will be elderly [1]. In addition, life expectancy was estimated as 80.3 years for men and 86.3 years for women in 2019, with the levels being higher than the average among countries in the Organization for Economic Co-operation and Development [2]. With the progression of aging and an increase in life expectancy, interest in maintaining the health of middle-aged and older adults has increased. This has also led to more emphasis being placed on oral health as a part of systemic health [3,4].

Generally, periodontal disease, as a chronic disease, and aging-related xerostomia, starts to appear in middle-aged adults, and the mismanagement of these conditions could lead to tooth loss [5]. Reduced mastication caused by severe tooth loss affects nutrient intake and brain cognitive functions, ultimately leading to reductions in muscular strength and physical function [6]. This negative impact may also extend to aesthetic aspects, inducing mental disorders due to problems in interpersonal relationships [7].

When an individual loses a tooth, a fixed prosthesis is required as a replacement, and implants are widely used in dental practice. The dental implant significantly contributes to improved oral health, as it restores the form and function as close to the natural tooth as possible, with the insertion of a fixture in the alveolar bone in the area of the lost tooth and applying an artificial tooth to the fixture [8]. Several previous studies have reported that the use of an implant tooth had a positive effect on the quality of life [9,10], increasing the interest in oral health and related facts. However, results vary according to individuals’ clinical and psychosocial characteristics [11].

Oral health influences the overall quality of life, which is closely associated with the performance of daily activities [12,13]. The two most important indicators of the level of physical functions in the older population are the activities of daily living (ADL), which are related to basic functions, such as getting dressed, taking a bath, or having a meal, and the instrumental ADL (IADL), which is related to more complex functions, such as phone use and meal preparation [14]. While several studies have reported the notable influence of ADL on the oral health-related quality of life in older adults [15,16], only a few studies in South Korea have examined the cause–effect relationship between ADL and the number of residual teeth as a key indicator of oral health.

Akifusa et al. [17] found a positive association between ADL and mastication in older adults, although no significant association was shown by the number of residual teeth. Nevertheless, a report indicated the positive role of the prosthetic restoration of lost teeth in maintaining or improving ADL [18], and another report revealed the association between tooth loss and xerostomia, among oral health problems, with disabilities and physical dysfunctions in older adults [19]. These studies [17,18,19] indicated that dental implants and number of teeth had an influence on ADL and IADL. Therefore, we hypothesized that, in middle-aged and older adults, a higher number of teeth corresponded to lower ADL and IADL levels.

The Korean Longitudinal Study of Aging (KLoSA) is conducted at the national level and is considered to have a high level of validity and reliability [20]. Therefore, using the panel data of the KLoSA, this study aimed to determine the number of teeth and ADL and IADL levels and examine the associations between these variables in middle-aged and older adults in South Korea.

## 2. Materials and Methods

### 2.1. Participants

The 2018 panel data of the KLoSA were used in this study [20]. The first KLoSA was conducted in 2006, and involved older adults aged ≥ 45 years (born before 1961). Since then, basic surveys have been conducted at 2-year intervals, and the 7th KLoSA was completed in 2018.

The samples were extracted through systematic sampling after selecting the participants using multistage stratified random sampling per region and household type. From the samples, 6925 participants aged ≥ 55 years and residing in a region other than Jeju Island in South Korea were selected to construct the panel data (https//survey.keis.or.kr/eng/klosa/klosa01.jsp accessed on 5 August 2021). This sample size was determined as sufficient for 10,000 residents over the age of 45 years in general housholds. Since the population aged 45 and over was 1.67 per household, the total number of households surveyed was 6000. To improve the convenience of conducting the survey and the representativeness of the sample, the number of households participating in the sample survey was set to 6 households per survey district, so the number of sample survey districts was 1000.

This study complied with the guidelines of the Helsinki Declaration and was approved by the Institutional Review Board of Dankook University (DKU No: 2020-08-013-EU002).

### 2.2. Variables

The main variables in this study were the number of natural teeth, number of implant teeth, and ADL and IADL levels.

Regarding this survey, trained investigators performed the computer-assisted personal interview (CAPI) based on the KLoSA standard protocol. All investigators participated in face-to-face collective education. Survey managers in all regions also participated in the investigators’ training. The investigators’ training instructor directly supervised the researcher in charge of KANTAR (https://www.kantarpublic.com/kr accessed on 20 September 2022). After consultation with the Korea Employment Information Service (KEIS) on the standard training plan that described the contents and training method, the investigators’ training was conducted based on the standard training plan. In the middle of the due diligence period, the researchers and investigators in charge of KEIS and KANTAR held a meeting to check their knowledge of the work, field information, and difficulties to better understand the investigation guidelines through mutual discussions [21].

The survey data were calibrated as follows:

When the CAPI survey was completed, the relevant data were uploaded. Supervisors and editors could access the program, and errors or omissions in the questionnaire were corrected via telephonic confirmation. To minimize errors in items wherein these occurred repeatedly, re-education was performed during the investigation process. In addition, data were finalized after the out-of-range values, branching between items, and logical errors were reviewed during the data cleaning process [21].

The number of natural teeth was first included as a variable in the 7th KLoSA. This was estimated by adding the baseline number of teeth (*n* = 28) to the number of remaining wisdom teeth (up to *n* = 4) and subtracting the numbers of implant or denture teeth and lost teeth. To measure the number of natural teeth, the numbers of implant teeth, teeth in dentures, missing teeth requiring implant or denture treatment, and remaining wisdom teeth, the question “How many of the following are applicable?” was asked. The detailed guidelines for answering this question were as follows:

− The maximum number of natural teeth a person possesses is 32, including wisdom teeth and, if there are no teeth, record “0”.
− For partial dentures, record the exact number of teeth; for full dentures, record maxillary or mandibular full dentures.
− If a tooth has roots, it is not a denture, and treatments such as amalgam, resin, gold teeth and treatments with remaining roots are considered natural teeth.
− Teeth that were intentionally extracted for therapeutic purposes, such as dentures and orthodontics, are not included in the number of missing teeth.

ADL and IADL were analyzed based on the questions of the KLoSA regarding the performance of daily activities. The ADL consisted of seven questions: getting dressed, washing the face/brushing the teeth/washing the hair, taking a bath or shower, having a meal, leaving the room, using the bathroom, and control of toilet. IADL consisted of 10 questions as follows: grooming, doing housework, meal preparation, doing laundry, traveling to local areas, using public transportation, purchasing things, money management, phone use, and taking medications on time. For each question, a score of 1 was given when assistance was partially or completely required in the performance of an activity, and a score of 0 was given when no assistance was required. The total score served as a numerical indicator, ranging between 0 and 7 for ADL and between 0 and 10 for IADL, with higher scores indicating a lower performance of daily activities [22].

The control variables included demographics (5 questions), health status (1 question), and health behavior (4 questions). For demographics, age was categorized as 55–64, 65–74, and ≥75 years; education level as elementary school or less, middle school, high school, and college or higher; sex as male and female; marital status as married, separated/divorced, and single; and health insurance as National Health Insurance (NHI) and medical aid. For health status, self-rated health was assessed using the 5-point Likert scale containing five response options: best (1 point); very good (2 points); good (3 points); moderate (4 points); and bad (5 points). In this study, “best”, “very good”, and “good” were integrated into “good” and recoded and analyzed [23]. For health behavior, working restriction and alcohol consumption were examined. The presence of dental implants was analyzed by recoding them as “yes” if they had more than one implant and “no” if they had no implants at all. The number of chronic diseases affecting an individual was measured by counting the diseases; these included hypertension, diabetes, cancer, chronic obstructive pulmonary disease, liver disease, cardiovascular disease, cerebrovascular disease, mental disease, and arthritis. If there was no relevant disease, the assigned classification was “0”, if there was one, the classification was “1”, and if there were two or more chronic diseases, the classification was “≥2” [23].

### 2.3. Statistical Analysis

SAS software (version 9.4; SAS Institute Inc., Cary, NC, USA) was used to produce descriptive statistics for all measured variables. As a result of the Kolmogorov–Smirnov analysis of the collected data, normality was ensured, and a parametric test was performed. To analyze ADL/IADL according to the number of teeth, the number of teeth was revised and analyzed as a categorical variable, classified into: “≤9”, “10–19”, and “≥20 “. For variations in the number of natural and implant teeth based on the general characteristics, the independent *t*-test and ANOVA were used. After controlling for all the general characteristics, multiple linear regression analysis was used to analyze the associations between the number of natural and implant teeth and ADL and IADL levels. The level of significance was set as 0.05.

## 3. Results

### 3.1. Number of Natural and Implant Teeth, ADL and IADL Levels, and General Characteristics

Table 1 presents the demographics of the study participants. Among the 6925 participants, the number of natural teeth was ≥ 20 in most participants (72.8%), ≤9 in 16.8% of them, and 10–19 in 10.4%. Moreover, the number of participants without implants (76.3%) was greater than the number with implants (23.7%). The age distribution was 66.7% for 55–64 years, 29.4% for 65–74 years, and 33.3% for ≥75 years. A greater number of participants had elementary school education or less (38.5%), and the percentage of men (42.3%) was lower than that of women (57.7%). Most participants were married (75.4%), and the response “No” to working restrictions and alcohol consumption was predominant, at 63.4% and 67.4%, respectively. Regarding health insurance, 95.5% had NHI, and 4.1% required medical aid. Most of the participants (90.7%) responded with “0” regarding the number of chronic diseases, with fewer having “1” (8.4%) or “≥2” (0.9%) chronic diseases. Concerning self-rated health, 44% reported moderate health, with 29.5% and 26.5% reporting good and bad health, respectively.

A higher number of natural teeth was significantly associated with a lower number of implant teeth, lower age, higher education level, male sex, absence of working restrictions, having NHI, and higher self-rated health (*p* < 0.001).

The number of implant teeth was higher in those aged 65–74 years (1.40) than in those aged 55–64 years (1.00) and ≥75 years (0.90) (*p* < 0.001). It also significantly increased as the education level increased (*p* < 0.001), and was higher in those without working restrictions (*p* = 0.009).

ADL/IADL levels were ADL (0.61)/IADL (1.56) in the participants with ≤9 teeth, ADL (0.40)/IADL (1.16) in those with 10–19, and ADL (1.10)/IADL (0.31) in the participants with ≥20 teeth. The ADL and IADL levels significantly worsened when the number of natural teeth decreased (*p* < 0.001).

ADL and IADL levels were significantly better in participants with implant teeth, a higher education level, no working restriction, alcohol consumption, NHI, lower number of chronic diseases, and higher self-rated health (*p* < 0.001). Although ADL was not significantly associated with marital status, IADL was significantly poorer for separated or divorced participants (*p* < 0.001). In addition, participants aged 65–74 years showed the poorest ADL levels, and those aged ≥75 years showed the poorest IADL levels, with statistical significance (*p* < 0.001).

### 3.2. Associations of ADL and IADL with the Number of Natural Teeth

Table 2 presents the results of the analysis of the associations between ADL and IADL with the number of natural teeth. Model 1 shows the result of the multiple linear regression analysis with adjustments for all variables except the number of implant teeth. No significant association was found between ADL and the number of natural teeth (*p* = 0.557). However, an increase in the IADL score by one unit was associated with a reduction in the number of natural teeth by –0.031 (95% confidence interval [CI] = from −0.043 to −0.018, *p* < 0.001).

**Figure 1:**
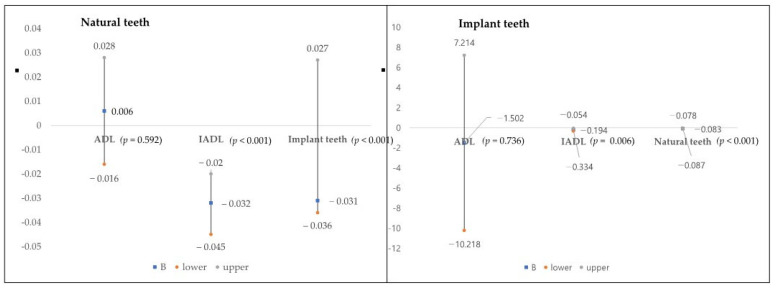
Association between ADL/IADL and natural teeth (**left**) and implant teeth (**right**). B, regression coefficient; vertical axis means the number of teeth. ADL, activities of daily living; IADL, instrumental activities of daily living.

Model 2 shows the result of the analysis with adjustments for all general characteristics and the number of implant teeth. Although an increase in the IADL score by one unit was associated with a reduction in the number of natural teeth by −0.032 (95% CI = −0.045 to −0.020, *p* < 0.001), no significant association was found between ADL and the number of natural teeth (*p* = 0.529). The number of natural teeth decreased by −0.031 (95% CI = −0.036 to −0.027, *p* < 0.001) when the number of implant teeth increased by one unit. Figure 1 (left) shows the effect size of the association between the number of natural teeth and ADL/IADL compared with the number of implant teeth (right).

### 3.3. Association of ADL and IADL with the Number of Implant Teeth

Table 3 presents the results of the analysis of the association between ADL and IADL with the number of implant teeth. Model 1 shows the results of the multiple linear regression analysis with adjustments for all variables except the number of natural teeth. No significant association was found between ADL or IADL and the number of implant teeth (*p* > 0.05).

Model 2 shows the result of the analysis with adjustments for general characteristics and the number of natural teeth. An increase in the IADL score by one unit was associated with a reduction in the number of implant teeth by −0.194 (95% CI = −0.334 to −0.054, *p* = 0.006), but no significant association was noted between ADL and the number of implant teeth (*p* > 0.05). The number of implant teeth decreased by −0.083 (95% CI = −0.087 to −0.078, *p* < 0.001) when the number of natural teeth increased by one unit. Figure 1 (right) shows the effect size of the association between the number of implant teeth and ADL/IADL compared with the number of natural teeth (left).

## 4. Discussion

With the recently increased emphasis on the rapid rate of aging as a social issue, the interest in oral health problems in older adults, in addition to other major health problems, has increased [24]. By analyzing the association between the number of teeth, as a key indicator of oral health status, and the performance of daily activities, this study aimed to identify health indicators in middle-aged and older adults. The 2018 panel data of KLoSA are highly reliable and, using these data, the associations between the number of natural teeth, number of implant teeth, and ADL and IADL levels were analyzed.

The participants in this study had a low number of natural teeth (*n* = 21). Based on the 6th Korea National Health and Nutrition Examination Survey (KNHANES), the number of natural teeth by age was 27.31 for 19–39 years, 25.17 for 40–64 years, and 16.64 for ≥65 years, showing a large margin of decrease in the number of teeth after the age of 65 years [25]. Tooth loss can be an early indicator of accelerated aging because it can induce some dysfunction in middle-aged and older adults [26].

While tooth loss has been reported to be associated with various internal and external factors of the oral cavity [27], its association with the performance of daily activities has not been adequately investigated. In our study, after adjusting for different variables, the number of natural teeth was significantly associated with the number of implant teeth, age, education level, sex, marital status, working restriction, alcohol consumption, health insurance, and self-rated health. Notably, analyzing the difference in the number of natural teeth based solely on the number of chronic diseases indicated that the number of teeth decreased as the number of chronic diseases increased, although no significant association was found in the regression analysis after adjusting for general characteristics.

Nevertheless, tooth loss has been reported to be associated with chronic diseases, the number of lost teeth and periodontal disease levels, increasing with the increased severity of diabetes and poorer diabetes control [28]. Periodontal disease and tooth loss may increase the levels of highly sensitive C-reactive protein, secreted during inflammation as a predictor of cardiovascular disease [29], and mortality due to cardiovascular disease and coronary artery disease has been shown to increase in proportion to the number of teeth [30]. The number of teeth is, therefore, a significant influencing factor in systemic and oral health. The result of this study, where the number of teeth decreased with increased age and self-rated health, was in agreement with a previous report on the influence of the current number of teeth on oral health-related quality of life [31].

In the 2005 KNHANES, 17.8% of older adults aged ≥ 65 years in South Korea required assistance when performing ADL, and 46.0% required assistance when performing IADL [32]. The ADL and IADL levels in our study significantly varied according to the presence of implant teeth, age, education, working restriction, alcohol consumption, health insurance, number of chronic diseases, and self-rated health. Poorer ADL and IADL levels were more common in older adults with lower education levels. This was in agreement with a previous study, which reported that there were more severe dysfunctions related to ADL and IADL for older adults in rural areas, in association with poverty, no NHI coverage, poorer self-rated health, and no participation in physical activity [33].

After adjusting for all variables, no significant association was found between ADL and the number of natural teeth. This finding is in line with that of a previous study reporting on the lack of a significant association between ADL and the number of natural teeth, despite their independent influence on health-related quality of life in super-aged individuals [17]. However, participants in the aforementioned study were limited to ≥ 85 years in a specific region, indicating some difference from the current study. Regarding IADL, a significant association was found, with the number of natural teeth decreasing by –0.031 with a one-unit increase in the IADL score. This finding is in line with that of a study that showed better IADL levels with an increase in the number of natural teeth, as the IADL score was higher for individuals without complete or partial dentures [34]. Our result is also in agreement with that of another study, which showed a decrease in IADL limitation by 3.1% when one more natural tooth was retained [35]. Takata et al. [36] conducted a study on older adults aged ≥ 80 years and found that functional dependency was 3.3-fold higher in individuals who could chew from five to nine types of food than in those who could chew 15 types of food, indicating the impact of mastication on ADL, although no significant association was shown between ADL and the number of healthy natural teeth. Nevertheless, based on the reported association between oral health status and functional dependency during daily activities in older adults, the association between the number of natural teeth, as a key oral health indicator, and the performance of daily activities may be inferred [37]. In addition, the generally reduced functional state may be attributed to the restriction of ADL, which follows the restriction of IADL. In the 2017 National Survey of the Living Conditions and Welfare Needs of Older Koreans, 16.6% of 10,299 older adults showed IADL restriction alone, while 8.7% had both ADL and IADL restrictions [38]. Therefore, it can be presumed that, compared to conventional ADL measurements, IADL/ADL measurements are more effective because they have a higher sensitivity for age and a higher level of hierarchy [39].

Analyzing the association between the number of implant teeth and ADL and IADL levels showed that, as with the number of natural teeth, no significant association was found regarding ADL. However, after adjustment for the number of natural teeth, there was a significant decrease in the number of implant teeth, by –0.194, as the IADL score increased by one unit. This agrees with a previous report, which showed higher IADL levels as mastication improved [40], and with another study conducted in South Korea that reported improvements in mastication and the Geriatric Oral Health Assessment Index as the number of fixed prostheses increased [41]. Dental prosthesis is a critical factor in the oral health of older adults who have an increased frequency of tooth loss with increasing age; lost teeth should be replaced with prostheses for functional restoration [42]. Based on this, the Ministry of Health and Welfare (https://www.mohw.go.kr/eng/ accessed on 20 September 2022) extended the scope of NHI coverage on dentures and implant teeth to include individuals aged ≥65 years. However, as prostheses cannot restore every function of the natural teeth, retention of the natural teeth is paramount [43]. To achieve this, an oral health management program for each phase of the life cycle should be developed for middle-aged and older adults [44].

IADL refers to the ability to respond to the social environment. Furthermore, ADL refers to the minimum ability that older people need for independent living [14]. Therefore, ADL/IADL is an index to measure the level of daily activities that are necessary for a family and social life [14]. In our study, the participants’ ADL levels were four times lower in the 10–19 teeth group and 6.1 times lower in the group of ≤9 teeth compared with the group of ≥20 teeth. The IADL levels were 3.7 times lower in the 10–19 teeth group and 5 times lower in the group of ≤9 teeth compared with the group of ≥20 teeth. In addition, as the number of teeth increased, the IADL levels significantly decreased (*p* < 0.001). Our results showed that the number of teeth and IADL levels in middle-aged and older adults were negatively related, suggesting that the number of teeth could be used as a biomarker of general health.

Our study is important, as there is a general lack of studies analyzing the association between the number of natural and implant teeth and ADL and IADL levels, although various studies have reported on the association between the number of natural teeth with geriatric diseases and oral health-related quality of life [45,46,47]. However, this study had some limitations. First, the KLoSA data started in 2006, but the variable measuring the number of teeth was from a cross-sectional study that was first conducted in 2018, and analyzing this in a longitudinal study was a limitation. Second, the CAPI was performed according to the standard protocol of KLoSA by trained investigators, but the clinical examination, such as determining the number of teeth, was not measured by a dental professional, so there is a limitation: there may be a bias due to measurement error. Third, it was impossible to control the factors affecting oral health because the various oral health behaviors of the participants in this study were not assessed.

Today, various intervention studies using natural products and oral education, including probiotics, are being conducted to promote oral health [48,49,50,51]. In the future, intervention studies related to oral health that can affect the improvement in ADL and IADL should be conducted, and it is necessary to expand these to continuous longitudinal studies. In addition, research should be conducted by examining oral health behaviors or other psychosocial factors that may affect the research results as confounding variables. Furthermore, it is necessary to develop strategies that enhance systemic health through oral health promotion, such as the maintenance of the number of natural teeth in middle-aged and older adults.

## 5. Conclusions

In Korean middle-aged and older adults, the level of oral health was shown to be low, with 21.2 natural teeth and 1.08 implant teeth. Herein, we first hypothesized that the higher the number of natural teeth, the lower the ADL and IADL levels. Therefore, in middle-aged and older adults, oral health promotion should be emphasized as a necessary measure to lead a healthy daily life. A regression analysis of the association between the number of natural and implant teeth and ADL and IADL levels revealed a significant negative association for IADL, but not for ADL. In other words, IADL levels improved as the number of natural or implant teeth increased, suggesting the need for various programs to maintain and manage the number of teeth in middle-aged and older adults in order to promote their health. Future longitudinal studies could examine the cause–effect relationship between the number of teeth and ADL and IADL levels.

## Figures and Tables

**Table 1:** General characteristics of the participants at baseline.

Variables	Total	Natural Teeth		Implant Teeth		ADL		IADL	
	*n* (%)	Mean ± SD	*p*-Value	Mean ± SD	*p*-Value	Mean ± SD	*p*-Value	Mean ± SD	*p*-Value
Natural teeth									
≤9	1161 (16.8)					0.61 ±1.8	<0.001	1.56 ± 3.1	<0.001
10–19	721 (10.4)					0.40 ± 1.5		1.16 ± 2.8	
≥20	5043 (72.8)					0.10 ± 0.8		0.31 ± 1.4	
Implant teeth			<0.001				<0.001		<0.001
No	5281 (76.3)	20.9 ± 10.8				0.30 ± 1.2		0.70 ± 2.2	
Yes	1644 (23.7)	22.1 ± 6.7				0.00 ± 0.5		0.20 ± 1.1	
Age (years)			<0.001		<0.001		<0.001		<0.001
55–64	2583 (37.3)	26.31 ± 5.3		1.00 ± 3.0		0.03 ± 0.4		0.10 ± 0.8	
65–74	2033 (29.4)	21.97 ± 8.9		1.40 ± 3.3		1.11 ± 0.8		0.32 ± 1.4	
≥75	2309 (33.3)	14.81 ± 11.2		0.90 ± 3.1		0.52 ± 1.7		1.42 ± 3.0	
Education level			<0.001		<0.001		<0.001		<0.001
Elementary school or less	2667 (38.5)	16.93 ±11.2		0.75 ± 2.7		0.42 ± 1.5		1.08 ± 2.7	
Middle school	1154 (16.7)	22.24 ± 9.3		1.16 ± 3.1		0.13 ± 0.9		0.42 ± 1.7	
High school	2225 (32.1)	24.22 ± 7.8		1.28 ± 3.4		0.07 ± 0.7		0.26 ± 1.3	
College or higher	879 (12.7)	25.14 ± 6.5		1.51 ± 3.5		0.09 ± 0.7		0.29 ± 1.3	
Sex			<0.001		0.071		0.559		0.048
Male	2931 (42.3)	21.42 ± 9.8		1.23 ± 3.3		0.19 ± 1.1		0.58 ± 2.0	
Female	3994 (57.7)	21.04 ± 10.1		0.98 ± 3.0		0.24 ± 1.2		0.62 ± 2.1	
Marital status			<0.001		0.083		0.734		<0.001
Married	5222 (75.4)	22.46 ± 9.2		1.18 ± 3.3		0.16 ± 1.0		0.43 ± 1.7	
Separated or divorced	1646 (23.8)	17.15 ± 11.2		0.78 ± 2.6		0.41 ± 1.5		1.15 ± 2.7	
Single	57 (0.8)	22.67 ± 9.7		1.07 ± 3.9		0.30 ± 1.3		0.88 ± 2.6	
Working restriction			<0.001		0.009		<0.001		<0.001
Yes	2536 (36.6)	17.69 ± 11.2		0.88 ± 2.9		0.54 ± 1.7		1.41 ± 3.0	
No	4389 (63.4)	23.23 ± 8.6		1.20 ± 3.2		0.03 ± 0.4		0.15 ± 0.9	
Alcohol consumption			0.037		0.080		<0.001		<0.001
Yes	2257 (32.6)	23.34 ± 8.5		1.25 ± 3.4		0.03 ± 0.4		0.16 ± 0.9	
No	4668 (67.4)	20.17 ± 10.5		1.00 ± 3.0		0.31 ± 1.3		0.82 ± 2.4	
Health insurance			<0.001		0.369		<0.001		<0.001
NHI	6640 (95.9)	21.40 ± 9.9		1.09 ± 3.1		0.20 ± 1.1		0.57 ± 2.0	
Medical aid	285 (4.1)	16.49 ± 11.6		1.05 ± 4.4		0.67 ± 1.9		1.49 ± 3.1	
Number of chronic diseases *			0.775		0.297		<0.001		<0.001
0	6282 (90.7)	21.36 ± 9.9		1.07 ± 3.1		0.19 ± 1.0		0.55 ± 1.9	
1	583 (8.4)	19.84 ± 10.6		1.27 ± 3.4		0.42 ± 1.5		1.06 ± 2.7	
≥ 2	60 (0.9)	18.30 ± 10.9		1.25 ± 2.7		0.92 ± 2.2		2.05 ± 3.7	
Self-rated health			<0.001		0.392		<0.001		<0.001
Good	2043 (29.5)	24.58 ± 7.6		1.10 ± 3.4		0.02 ± 0.3		0.08 ± 0.7	
Moderate	3048 (44.0)	21.97 ± 9.3		1.16 ± 3.0		0.03 ± 0.4		0.19 ± 1.0	
Bad	1834 (26.5)	16.17 ± 11.4		0.95 ± 3.1		0.75 ± 2.0		1.89 ± 3.4	
All	6925 (100.0)	21.20 ± 10.0		1.08 ± 3.1		0.22 ± 1.1		0.61 ± 2.0	

* Hypertension, diabetes, cancer, chronic obstructive pulmonary disease, liver disease, cardiovascular disease, cerebrovascular disease, and arthritis; *p*-values were calculated with the independent *t*-test or one-way analysis of variance (ANOVA) test at α = 0.01. ADL, activities of daily living; IADL, instrumental activities of daily living; NHI, National Health Insurance; SD, standard deviation

**Table 2:** Association between ADL/IADL and natural teeth.

Variables	Model 1			Model 2		
	B	95% CI	*p*-Value	B	95% CI	*p*-Value
ADL	0.007	−0.016, –0.029	0.557	0.006	−0.016, 0.028	0.592
IADL	−0.031	−0.043, –0.018	<0.001	−0.032	−0.045, −0.020	<0.001
Implant teeth				−0.031	−0.036, 0.027	<0.001
Age (years)						
55–64	1			1		
65–74	−0.112	−0.135, 0.089	<0.001	−0.095	−0.117, 0.073	<0.001
≥75	−0.399	−0.431, 0.367	<0.001	−0.391	−0.423, −0.360	<0.001
Education level						
Elementary school or less	−0.136	−0.170, −0.103	<0.001	−0.160	−0.193, −0.127	<0.001
Middle school	−0.047	−0.106, −0.042	<0.001	−0.058	−0.090, −0.027	<0.001
High school	−0.034	−0.067, −0.013	0.004	−0.039	−0.065, −0.012	0.004
College or higher	1			1		
Sex						
Male	1					
Female	0.048	0.026, −0.069	<0.001	0.049	0.028, 0.070	<0.001
Marital status						
Married	1			1		
Separated or divorced	−0.043	−0.071, −0.016	<0.001	−0.048	−0.075, −0.022	<0.001
Single	−0.013	−0.110, 0.085	0.686	0.010	−0.105, −0.084	0.831
Working restriction						
Yes	−0.044	−0.068, −0.020	<0.001	−0.052	−0.075, −0.029	<0.001
No	1			1		
Alcohol consumption						
Yes	1			1		
No	−0.010	−0.031, −0.011	0.037	−0.014	−0.035, −0.006	0.175
Health insurance						
NHI	1	Reference		1		
Medical aid	−0.079	−0.141, −0.018	0.017	−0.071	−0.130, −0.012	0.019
Number of chronic diseases *						
0	1			1		
1	0.007	−0.029, 0.043	0.983	0.014	−0.021, 0.048	0.438
≥2	0.026	−0.088, 0.139	0.694	0.026	−0.085, 0.137	0.350
Self-rated health						
Good	1			1		
Moderate	0.104	0.072, 0.137	<0.001	0.097	0.065, 0.129	<0.001
Bad	0.091	0.060, 0.121	<0.001	0.087	0.057, 0.117	<0.001

* Hypertension, diabetes, cancer, chronic obstructive pulmonary disease, liver disease, cardiovascular disease, cerebrovascular disease, and arthritis. All models were adjusted for all other variables except the target variable; Model 1 was adjusted for all variables except the number of implant teeth; Model 2 was adjusted for all general characteristics and the number of implant teeth; *p*-values were calculated using multiple regression analysis at α = 0.01. ADL, activities of daily living; CI, confidence interval; IADL, instrumental activities of daily living; NHI, National Health Insurance.

**Table 3:** Association between ADL/IADL and implant teeth.

Variables	Model 1			Model 2		
	B	95% CI	*p*-Value	B	95% CI	*p*-Value
ADL	−0.116	−0.398, 0.166	0.419	−1.502	−10.218, 7.214	0.736
IADL	−0.076	−0.169, 0.017	0.110	−0.194	−0.334, −0.054	0.006
Natural teeth				−0.083	−0.087, −0.078	<0.001
Age (years)						
55–64	1			1		
65–74	0.550	0.392, 0.709	< 0.001	−0.215	−0.323, −0.106	<0.001
≥75	0.533	0.338, 0.727	< 0.001	−0.636	−0.786, −0.486	<0.001
Education level						
Elementary school or less	−0.856	−1.083, −0.629	< 0.001	−1.458	−1.669, −1.248	<0.001
Middle school	−0.396	−0.603, −0.190	< 0.001	−0.680	−0.836, −0.523	<0.001
High school	−0.186	−0.349, −0.022	0.026	−0.300	−0.409, −0.192	<0.001
College or high	1			1		
Sex						
Male	1			1		
Female	0.094	−0.055, 0.244	0.216	0.347	0.238, 0.457	<0.001
Marital status						
Married	1			1		
Separated or divorced	−0.244	−0.450, −0.038	0.020	−0.546	−0.739, −0.352	<0.001
Single	0.064	−0.672, 0.799	0.865	0.281	−0.110, 0.671	0.159
Working restriction						
Yes	−0.193	−0.356, −0.030	0.020	−0.582	−0.738, −0.426	<0.001
No	1			1		
Alcohol consumption						
Yes	1			1		
No	−0.161	−0.304, −0.018	0.027	−0.329	−0.435, −0.224	<0.001
Health insurance						
NHI	1			1		
Medical aid	0.004	−0.389, 0.397	0.984	0.172	−0.003, 0.346	0.054
Number of chronic diseases *						
0	1			1		
1	0.166	−0.030, 0.362	0.096	−0.117	−0.285, 0.050	0.170
≥2	0.098	−0.516, 0.712	0.755	−0.076	−0.716, 0.563	0.815
Self-rated health						
Good	1			1		
Moderate	−0.205	−0.407, −0.002	0.047	0.142	−0.026, 0.311	0.098
Bad	−0.157	−0.337, 0.023	0.087	−0.102	−0.267, 0.062	0.222

* Hypertension, diabetes, cancer, chronic obstructive pulmonary disease, liver disease, cardiovascular disease, cerebrovascular disease, and arthritis. All models were adjusted for all other variables except the target variable; Model 1 was adjusted for all variables except the number of natural teeth; Model 2 was adjusted for all general characteristics and the number of natural teeth; *p*-values were calculated using multiple regression analysis at α = 0.01. ADL, activities of daily living; CI, confidence interval; IADL, instrumental activities of daily living; NHI, National Health Insurance.

## Data Availability

The data of the KLoSA are publicly available on the KLoSA website (https://survey.keis.or.kr/klosa/klosa01.jsp accessed on 5 August 2021). The data presented in this study are available on request from the corresponding author.
